# *Bifidobacterium animalis* subsp. *lactis* BB-12 Has Effect Against Obesity by Regulating Gut Microbiota in Two Phases in Human Microbiota-Associated Rats

**DOI:** 10.3389/fnut.2021.811619

**Published:** 2022-01-10

**Authors:** Kemin Mao, Jie Gao, Xianghong Wang, Xiyu Li, Shuo Geng, Tuo Zhang, Faizan Ahmed Sadiq, Yaxin Sang

**Affiliations:** ^1^Department of Food Science and Technology, Hebei Agricultural University, Baoding, China; ^2^School of Food Science and Technology, Jiangnan University, Wuxi, China

**Keywords:** *Bifidobacterium animalis* subsp. *lactis* BB-12, gut microbiota, obesity, *Prevotella*, enterotypes

## Abstract

*Bifidobacterium animalis* subsp. *lactis* BB-12 (BB-12) is an extensively studied probiotics species, which has been reported to improve the human gut microbiota. This study aimed to confirm the effects of BB-12 on high-fat diet (HFD)-induced gut microbiota disorders. The probiotic BB-12 was consumed by human microbiota-associated rats and changes in gut microbiota were compared using next generation sequencing of the fecal samples collected from the normal chow group, the HFD group, and the BB-12-supplemented group. The enterotypes switched from *Prevotella* dominant to *Akkermansia* dominant as a result of switching diet from normal chow to HFD. BB-12 conferred protection on the gut microbiota composition of the rats by increasing the abundance of *Prevotella* and decreasing the abundance of *Clostridium, Blautia*, and *Bacteroides* in 0–3 weeks. In addition, *Prevotella*-dominant enterotype was maintained, which provides improve obesity effects. A decrease in body weight and the *Firmicutes/Bacteroidetes* ratio were also observed at week 3. While in 4–8 weeks, the enrichment of short-chain fatty acids-producing bacteria such as *Eubacterium* and *Parabacteroides* and probiotics such as *Bifidobacterium* was observed. The results revealed that BB-12 against obesity by regulating gut microbiota in two phases. After a short-term intervention, BB-12 supplementation suppressed the transition from the healthy to obesity state by protecting *Prevotella*-dominant enterotype, whereas after a long-term intervention, BB-12 ameliorates obesity by enriching beneficial bacteria in the gut.

## Introduction

Obesity is one of the major health crises, especially among children and adults, at the global level. Obese people are at increased risk of morbidity and obesity-related comorbidities such as diabetes, metabolic syndrome, and cardiovascular diseases (1). Human gut microbiota is considered as a key element of good health, as it has marked influence on immune homeostasis and body physiology and functionality. There are multitude of studies, which have provided evidence of its correlation with health and diseases, especially obesity and metabolic disorders (2, 3). Many studies have shown dysbiosis of the human gut microbiota in obese individuals (4). In many previous studies, when germ-free (GF) mice were inoculated with feces or the microbiota of obese human subjects, they developed symptoms such as increased weight and many obesity-associated metabolic phenotypes (5, 6). Dietary elements have the ability to alter the community structure of gut microbiota. A complicated “three-way” connection among gut microbiota, host health, and the environmental inputs has been proposed. Dietary pattern is one of the environmental inputs modifying the composition of the gut microbiota (7). A study showed that dietary change may alter the structure of human gut microbiota up to 57% (8). High-fat diet (HFD) may exacerbate obesity by changing the structure of gut microbiota and by promoting obesogenic bacteria (9, 10); dietary intervention with probiotics may attenuate adipocyte size in mice fed a HFD and control weight gain (11). Thus, improving the gut microbiota is an effective strategy for the prevention and management of diet-induced obesity.

The use of probiotics is one of the main dietary strategies to modulate gut microbiota, as they are generally recognized as safe for human use (12, 13). Most studies have shown that probiotics maintain the intestinal microbiome homeostasis and redress particular disease states associated with microbiota dysbiosis (3, 14). Members of the genus, *Bifidobacterium*, are widely used as probiotics because of their ability to prevent and treat a wide spectrum of animal and human gastrointestinal disorders such as colonic transit disorders and intestinal infections (15). *Bifidobacterium animalis* subsp. *lactis* BB-12 (BB-12) is the most documented probiotic species among all the members of the genus *Bifidobacterium* because they exhibit excellent gastric acid and bile tolerance, contain bile salt hydrolases, and have strong mucus adherence properties—all the valuable probiotic characteristics (16). BB-12 has been used in a plethora of successful clinical trials on a range of people including infants, children, adults, and elderly and a lot of beneficial effects have been reported such as management of infantile colic (17), improvement of immune system (18), reduction of the risk of infections in early childhood (19), and balancing of the disturbed gut microbiota as a result of severe acute malnutrition (20). Besides, BB-12 has been used in dietary supplements, fermented milk products, and infant formula worldwide (21). There are only a few studies, which have reported the effect of BB-12 in ameliorating obesity. The effects of many other probiotic strains on obesity have been verified and reported; for instance, the influence of probiotic supplementation on reducing body weight, body mass index (BMI), and fat percentage (22–25). Some special probiotics belonging to the genus *Lactobacillus* [such as *L*. *casei* strain *Shirota* (LAB13), *L*. *gasseri, L*. *rhamnosus*, and *L*. *plantarum*] and *Bifidobacterium* (such as *B*. *infantis, B*. *longum*, and *B*. *breve* B3) species have been successfully used to improve obesity and its medical complications in animals (23). *Lactobacillus rhamnosus* GG supplementation has been reported to reduce the body weight gain in children (26). These studies support that probiotics may ameliorate obesity as a safe and effective methods.

In this study, female GF Sprague-Dawley (SD) rats were used to build human microbiota-associated (HMA) rats, which were used to explore changes in gut microbiota following a feeding response to HFD. The impact of administration of BB-12 on obesity was studied as a dietary intervention strategy. 16S ribosomal RNA (16S rRNA) sequencing was used to evaluate the changes of gut microbiota composition. The purpose of this study is to investigate how BB-12 supplementation could further influence host obesity via modification of the gut microbiota structure and composition.

## Materials and Methods

### BB-12 Preparation

The probiotic strain BB-12 was purchased from Chr Hansen, Denmark and prepared by suspending lyophilized powdered bacteria in sterile water. All the suspensions of BB-12 for oral gavage were freshly prepared.

### Human Fecal Microbiota Preparation

Fresh fecal samples were collected from a 49-year-old female volunteer after receiving a written informed consent. The volunteer was a metabolically healthy obese person with BMI 29.3 kg/m^2^ (27); in addition, the volunteer had no special diet requirements or lifestyle, normal blood lipid profile, normotension, euglycemia and no chronic diseases, and had not taken any antibiotics/probiotics for the last at least 3 months. Then, first feces of the volunteer were collected in the morning and suspended into 0.1 M phosphate-buffered saline (PBS) buffer (pH 7.2), before it was fed to rats.

### Rat Models and Sample Collection

**Figure 1:**
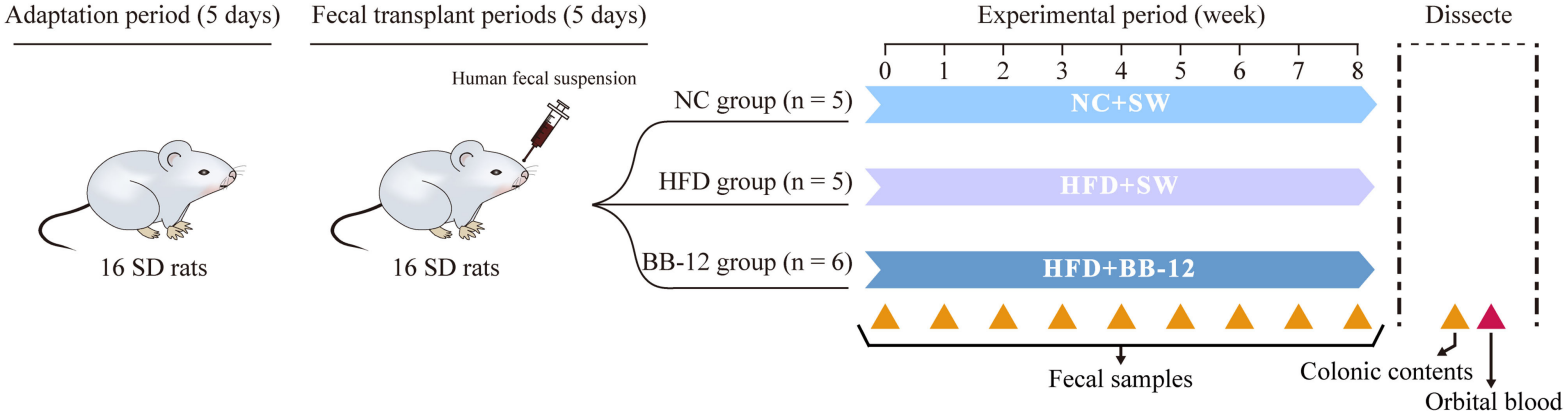
Group information of HMA-SD rat models. After 5 days of adaptation, human fecal suspension was intragastrically administered every 2 days three times to build the HMA-rats model. Then, the 16 HMA-SD rats were randomly divided into 3 groups for a 8-week trial. The normal chow (NC) group, 8 weeks of NC and daily administration of sterile water gavage, as negative control; the high-fat diet (HFD) group, 8 weeks of HFD and sterile water gavage; the BB-12 group, 8 weeks of HFD and BB-12 gavage. HMA-SD, human microbiota-associated-Sprague-Dawley; SW, sterile water; TKM, Tibet kefir milk; 16S rRNA, 16S ribosomal RNA. Yellow triangle up solid, sampling sites for 16S rRNA sequencing; red triangle up solid, sampling sites for serum lipid profile.

All the experimental procedures involving animals in this study were performed according to the principles and guidelines established by the Centers for Disease Control and Prevention, China. GF female SD rats (*n* = 16, 8 weeks old) were purchased from the Institute of Laboratory Animal Science of Chinese Academy of Medical Sciences (CAMSs) and Peking Union Medical College (PUMC) and raised in sterile microisolators. After a 5-day acclimatization period, the fecal suspension was administered via oral gavage every 2 days for three times to build HMA-rats model. Then, the rats were randomly divided into 3 groups: the normal chow (NC) group (*n* = 5), the HFD group (*n* = 5), and the group receiving probiotic supplement (BB-12) (*n* = 6). HFD was D12492 (containing 60% fat; Research Diets Incorporation, New Brunswick, NJ, USA), while the NC was D1245B (containing 10% fat, Research Diets Incorporation) and the gavage volume of BB-12 was 9 × 10^7^ colony-forming unit (CFU)/kg·body weight (bw). More details are shown in Figure 1.

We recorded the body weight and collected feces once a week during this study. All the rats were sacrificed with lethal dose of diethyl ether at the end of 8 weeks and orbital blood samples and colonic contents were collected. All the samples were stored at −80°C until further use, except orbital blood. The blood was centrifuged (3,000 rpm, 20 min) to obtain serum and serum levels of triglyceride (TG), total cholesterol (TC), low-density lipoprotein cholesterol (LDL-C), and high-density lipoprotein cholesterol (HDL-C) were determined using an automatic blood chemical analyzer, Roche Applied Science, Rotkreuz, Switzerland (COBAS INTEGRA 800, Roche).

### Analysis of Gut Microbiota by Next Generation Sequencing (NGS)

Microbial DNA was extracted from fecal samples and colonic contents using the DNA Stool Mini Kit (BGI Corporation Ltd., Beijing, China). The V4 hypervariable region of the bacterial 16S rRNA gene was amplified using aliquots of the isolated DNA from each sample using the primers 515F (GTGCCAGCMGCCGCGGTAA) and 806R (GGACTACHVGGGTWTCTAAT) on an Illumina MiSeq PE250 Platform (Illumina, San Diego, United States). Paired-end reads were first merged to tags and then all the tags were clustered to operational taxonomic unit (OTU) at the 97% sequence similarity level. OTUs were taxonomically classified by the Ribosomal Database Project (RDP) classifier (2.2), which was trained on Greengenes database using 0.18 confidence values as the cutoff.

### Statistical Analyses

Statistical analyses were performed using R programming language. The methods described by Arumugam et al. (28) were used for enterotypes (ETs) analysis of gut microbiome. Bacterial functional gene redundancies were predicted with Tax4Fun2 with R package (29). The linear discriminant analysis effect size (LEfSe) algorithm was used to identify the differential abundance of bacterial taxa. The differential Kyoto Encyclopedia of Genes and Genomes (KEGG) pathway enrichment analysis was performed with the online interface Galaxy (http://huttenhower.sph.harvard.edu/galaxy). Co-occurrence relationships between the gut microbiota and the different KEGG orthology or gene pathways were determined based on the Pearson's correlation coefficients. A parametric test (one-way ANOVA followed by the Tukey's *post-hoc* test) was applied when normality assumptions and homogeneity of variance were satisfied. Non-parametric data were assessed using the Wilcoxon rank-sum test. *p* < 0.05 was considered as statistically significant (^*^p < 0.05, ^**^p < 0.01, ^***^p < 0.001). Graphic presentations were generated using the R package “ggplot 2” (30).

## Results

### Effect of BB-12 Supplementation on the BW and Blood Lipids

The BW gains of the rats in the HFD group (364.2 ± 11.1 g) were significantly higher than the BW of the rats in the NC group (307.6 ± 12.6 g), which showed that the model of obesity was successfully constructed (Supplementary Figure 1A). The BW of BB-12-fed rat (355.5 ± 4.8 g) was similar to the body weight of HFD rats at week 8. However, at the third week, the body weight was decreased in BB-12-fed rats (298.0 ± 4.7 g) compared with the rats in the HFD group (317.8 ± 8.1 g). As shown in Supplementary Figures 1B–E, there was no significant difference in blood lipid profile as a result of either feeding with a HFD or NC, with or without BB-12.

### Effect of BB-12 Supplementation on the Community Structure of Gut Microbiota

16S ribosomal RNA gene sequencing of 159 samples (143 fecal samples and 16 colonic samples) from 16 SD rats was performed. Species richness and alpha diversity were determined with OTUs, which were identified in the fecal samples (Supplementary Figure 2). Observed species and Chao1 indices reflect species richness, while Shannon and Simpson indices represent microbial alpha diversity. The Wilcoxon analysis revealed no significant difference in alpha diversity among the three groups after 8 weeks of feeding period. However, the alpha diversity increased in the BB-12-fed rat group compared with the HFD group at the third week.

**Figure 2:**
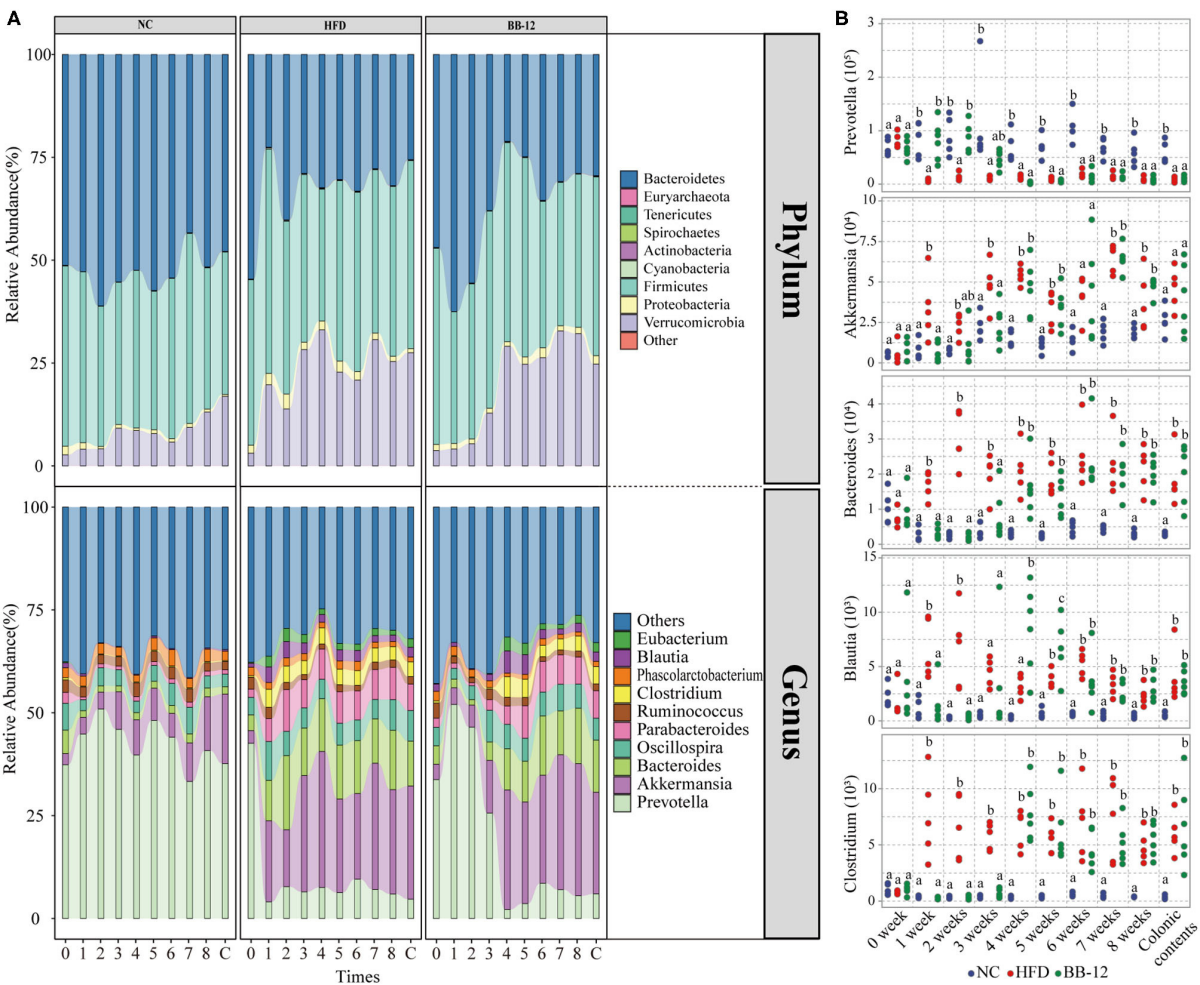
Gut microbiota taxonomic profiles. **(A)** Relative abundances of microbial composition at the phylum level and relative abundances of top 10 genera; 0–8 represents 0–8 weeks and C represents colonic content samples. **(B)** Absolute abundances of microbial taxa (genera level) compared between the NC, HFD, and BB-12 groups. Different letters (a or b) above the box indicate significant difference (*p* < 0.05) or (*p* < 0.01).

**Figure 3:**
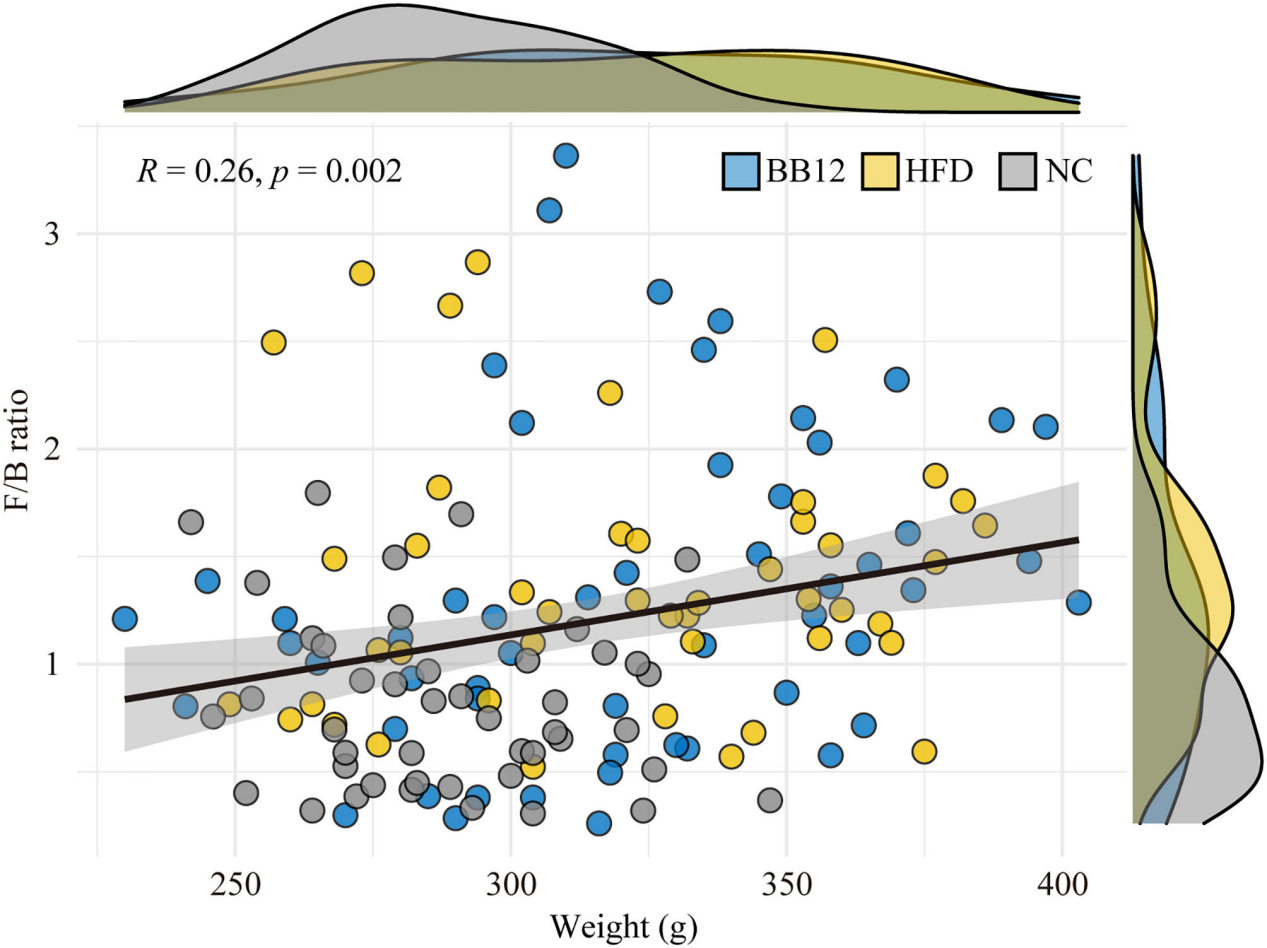
Correlation analysis between body weight and *Firmicutes/Bacteroidetes* ratio. The graph shows arbitrary data for the NC group (gray circles and density curves), the HFD group (light yellow circle and density curves), and the BB-12 group (light blue circle and density curves).

Relative abundances of the gut microbiota at the phylum level are given in Figure 2A. A total of 9 bacterial phyla were identified by NGS. Among them, *Bacteroidetes, Firmicutes*, and *Verrucomicrobia* were the three most dominant phyla in all the groups. In the NC group, all the three dominant phyla were relatively stable during the feeding period. In the HFD group, *Verrucomicrobia* increased in the first week, while *Bacteroidetes* decreased and then remained relatively stable in the following feeding period. In the BB-12-fed rats group, *Bacteroidetes* showed an increasing trend in the first week and then started decreasing until 4 weeks and became stable afterward. *Verrucomicrobia* increased in the first 4 weeks and remained stable during the following feeding periods. The correlation analysis between BW and *Firmicutes/Bacteroidetes* (F/B) ratio is shown in Figure 3, where a weak but significantly positive correlation was found between BW and F/B ratio.

At the genus level (Figure 2A), a total of 68 bacteria genera were identified in all the groups. Among them, *Prevotella, Akkermansia, Bacteroides, Oscillospira, Parabacteroides, Ruminococcus, Clostridium, Phascolarctobacterium, Blautia*, and *Eubacterium* were the top 10 dominant genera in all the groups. In the NC group, bacterial community composition of all the top 10 genera was relatively stable during the feeding period. In the HFD group, changes in bacterial community structure were noticed only a week following feeding. For example, the abundance of *Bacteroides* and *Akkermansia* increased in the first week and then remained stable at a high level. An opposite trend was noticed for the abundance of *Prevotella*, which decreased in the first week and then remained stable at the same low level over the following feeding weeks. In the group, where rats were fed with the BB-12 group, the abundance of *Prevotella* increased in the first week and then started decreasing until 4 weeks and remained stable whereas the abundance of *Akkermansia, Bacteroides, Parabacteroides, Blautia*, and *Clostridium* increased after 4 weeks of BB-12 supplementation.

The absolute abundance analysis (Figure 2B) of bacterial community composition showed distinct abundance profiles of rats as a result of HFD and BB-12 supplementation. Compared with the NC group rats, the absolute abundance of *Clostridium, Akkermansia, Blautia*, and *Bacteroides* increased in the HFD group rats, while the abundance of *Prevotella* decreased in the HFD group rats. Compared with the HFD group rats, changes of gut microbiota in the BB-12-fed rats group could be divided into two parts: (1) period from weeks 1 to 3, in which BB-12 supplementation resulted in a decrease in the absolute abundance of *Clostridium, Akkermansia, Blautia*, and *Bacteroides* whereas it resulted in an increase in the abundance of *Prevotella* and (2) period from weeks 4 to 8, in which there was no significant difference between the HFD group and BB-12 group in terms of these bacterial groups.

**Figure 4:**
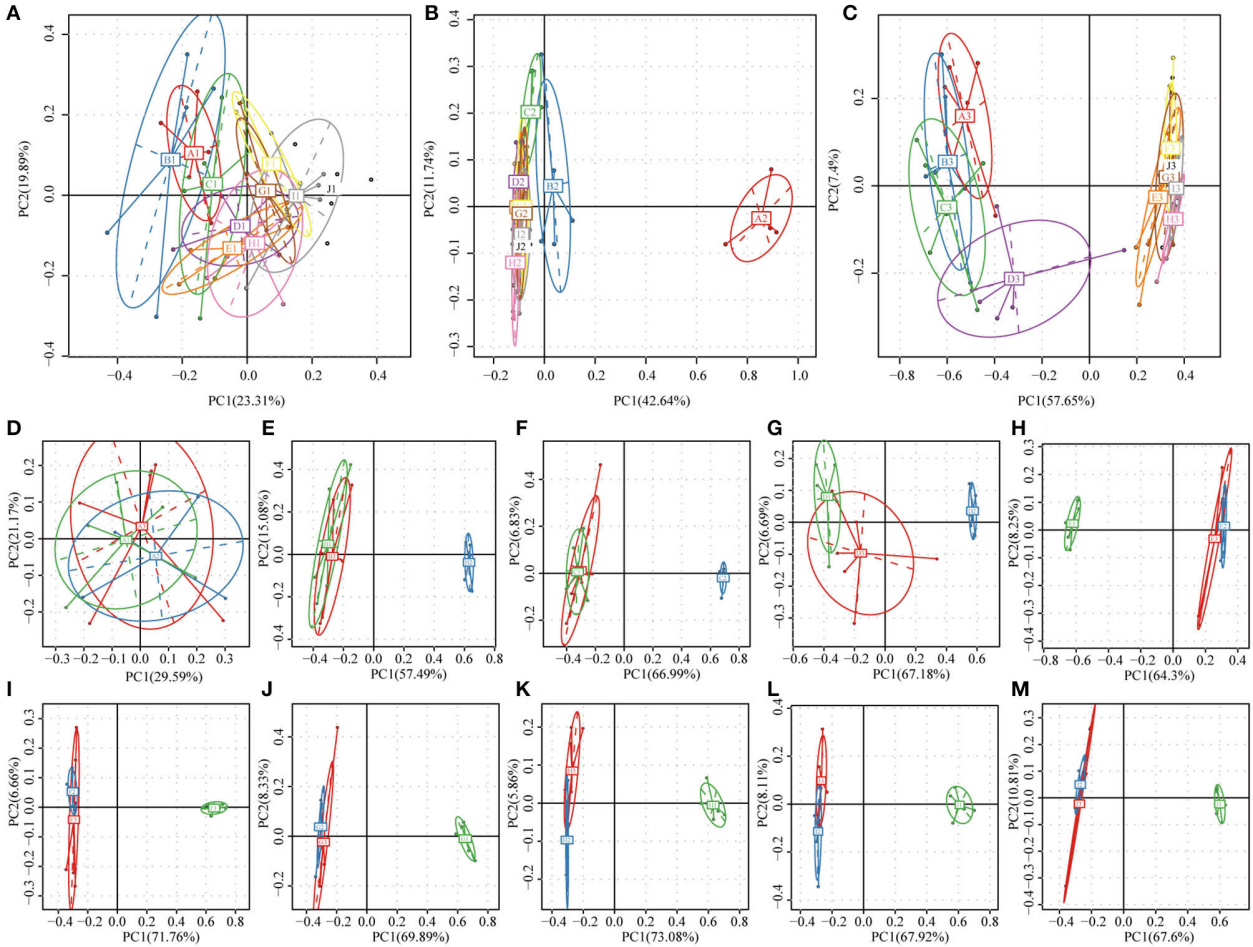
Principal component analysis (PCA) of bacterial community composition based on relative abundances of 16S rRNA from 159 samples. **(A–C)** Show the NC, HFD, and BB-12 group, respectively; letters A–I in the figures represent the fecal samples from 0 to 8 weeks and J represents colonic content samples. **(D–L)** represent the fecal samples at 0–8 weeks, respectively and **(M)** represented the colonic contents samples; the numbers 1–3 in **(D–M)** represent the NC, HFD, and BB-12 group, respectively.

Principal component analysis (PCA) is shown in Figure 4. The gut microbiota communities of the rats belonging to the NC group remained relatively stable during the 8 weeks feeding period (Figure 4A). In the HFD group, samples from all the 8 weeks clustered together except for 0 week before feeding test (Figure 4B). In the BB-12-fed rats group (Figure 4C), samples could be divided into 4 clusters from 0 to week 3 and then clustered together in one group from weeks 4 to 8. Weekly distributions between 3 groups were shown in Figures 4E–M. All the samples were clustered together at 0 week (Figure 4D). In all the 8 weeks of the feeding period, rats of the HFD and NC groups could be divided into two clusters. Bacterial absolute abundance pattern in the BB-12-fed rats group was closely related to the NC group rats from weeks 1 to 3 whereas it was close to bacterial absolute abundance pattern in HFD rats from weeks 4 to 8.

**Figure 5:**
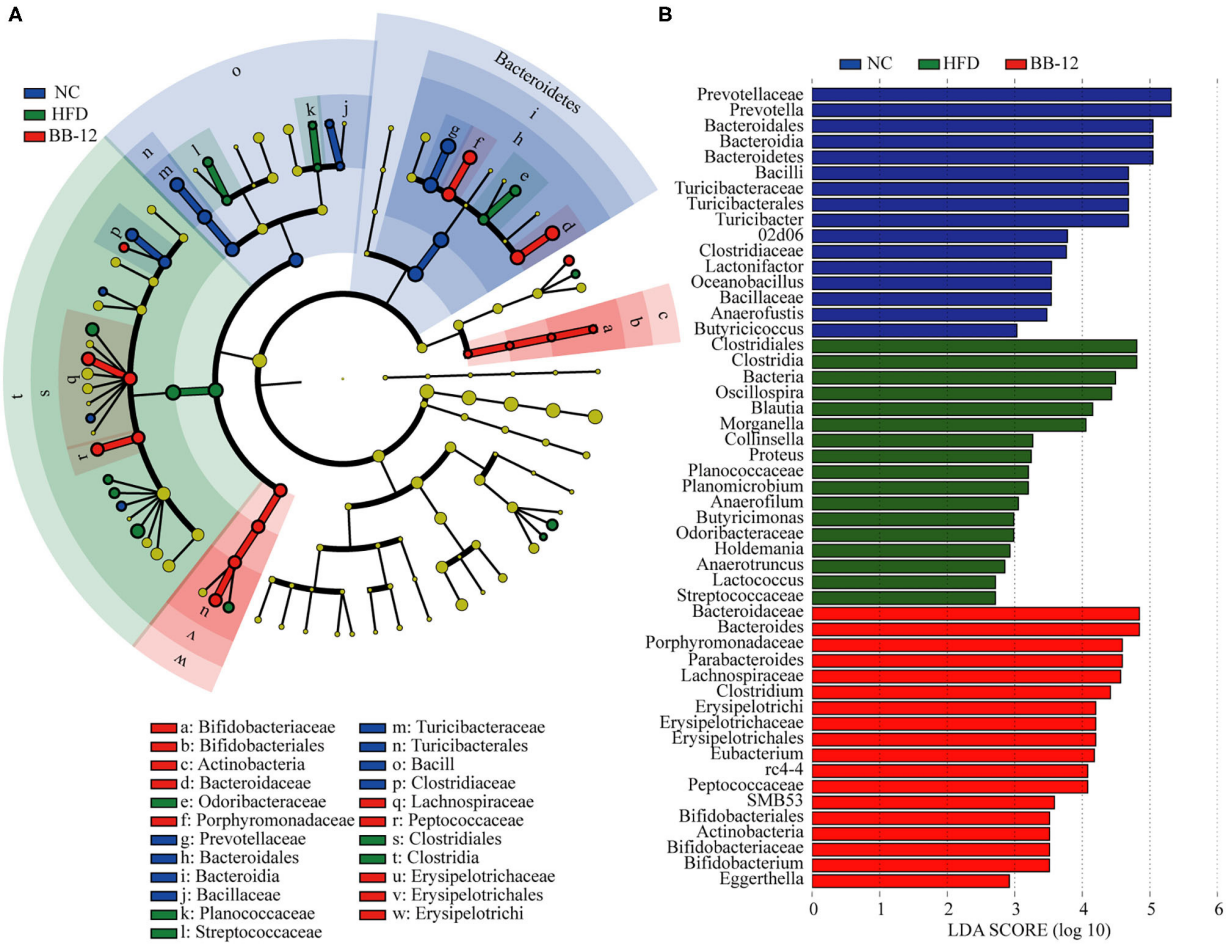
Linear discriminant analysis (LDA) effect size (LEfSe) analysis of bacterial community composition based on relative abundances of 16S rRNA from 16 colonic contents samples. **(A)** LEfSe cladogram representing different abundant taxa; and **(B)** LDA scores as calculated by LEfSe analysis. Only taxa with LDA scores of more than 2 were presented.

Linear discriminant analysis effect size of colonic contents microbiota is shown in Figure 5. At the genus level, *Anaerofustis, Butyricicoccus, Lactonifactor, Oceanobacillus, Prevotella*, and *Turicibacter* were enriched in rats of the NC group. *Anaerofilum, Anaerotruncus, Blautia, Butyricimonas, Collinsella, Holdemania, Lactococcus, Morganella, Oscillospira, Planomicrobium*, and *Proteus* were enriched in the HFD group whereas *Bacteroides, Bifidobacterium, Clostridium, Eggerthella, Eubacterium*, and *Parabacteroides* were enriched in rats of the BB-12 group.

### Effect of BB-12 Supplementation on ETs

**Figure 6:**
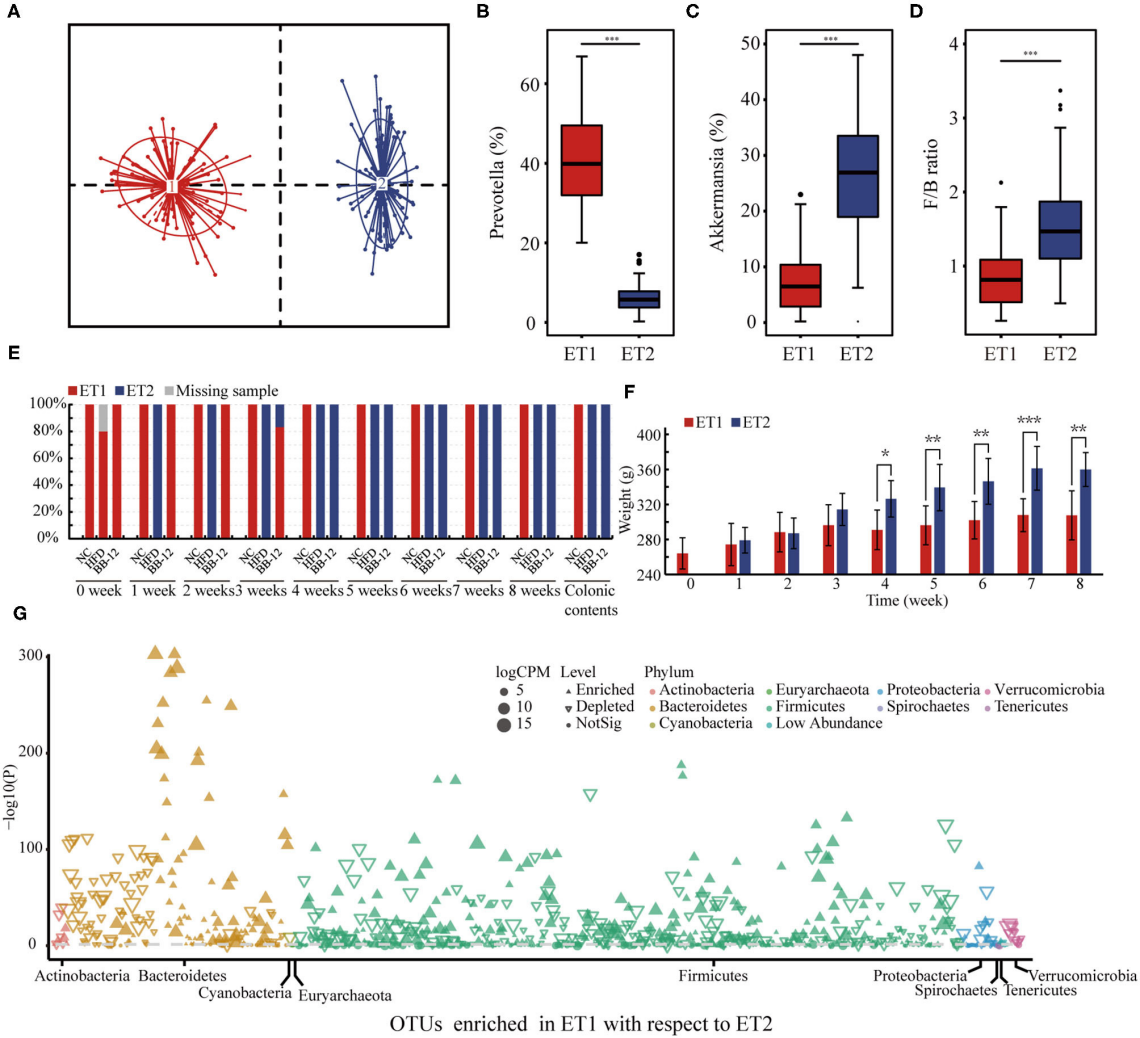
Enterotype (ET) analysis based on the genus-level bacterial composition of the gut microbiota. **(A)** 159 samples were divided into two different ETs; **(B,C)** represent the relative abundance of *Prevotella* and *Akkermansia* in each ETs, respectively; **(D)** represents the *Firmicutes/Bacteroidetes* ratio into two different ETs; **(E)** distribution of ETs by each group; **(F)** represents the body weight in different ETs; **(G)** Manhattan plot showing OTUs being enriched in ET1 or ET2; each dot or triangle represents a single OTU. OTUs enriched in ET1 or ET2 are represented by filled or empty triangles, respectively (FDR adjusted *p* < 0.05). OTUs are arranged in taxonomic phylum. OTUs, operational taxanomic units; FDR, false discovery rate; CPM, counts per million; ET1, enterotype 1; ET2, enterotype 2. ^*^p < 0.05; ^**^p < 0.01; ^***^p < 0.001.

All the 159 samples were divided into 2 clusters: enterotype 1 (ET1) and enterotype 2 (ET2) (Figure 6A). ET1 was dominated by *Prevotella* as the most enriched genus (Figure 6B) and included 77 samples (Figure 6E). As shown in Figure 6C, *Akkermansia* was the core genus in ET2, which included 82 samples (Figure 6E). All the samples in the NC group belonged to ET1. In the HFD group, rats could first be characterized as belonging to ET1 at week 0 and then to ET2 at week 1. In the BB-12-fed rats group, rats belonged to ET1 at weeks 0 to 3 could be characterized as belonging to ET2 at week 4. F/B ratio of the ET2 groups was higher than the samples in the ET1 group (Figure 6D). The BW of rats in the two ET groups was analyzed and no significant difference in first 4 weeks was noticed. The BW of rats in the ET2 group was higher than the ET1 group from weeks 4 to 8 (Figure 6F). Manhattan plots were used to examine differences between ET1 and ET2 at the OTU level (Figure 6G). Compared with ET2, 308 OTUs were enriched in ET1 and 283 OTUs were diminished and the differences in OTUs mainly spanned 9 phyla, with the phyla *Bacteroidetes* and *Firmicutes* accounting for the majority of the differences.

### Effect of BB-12 Supplementation on Functional Properties of Bacterial Community

**Figure 7:**
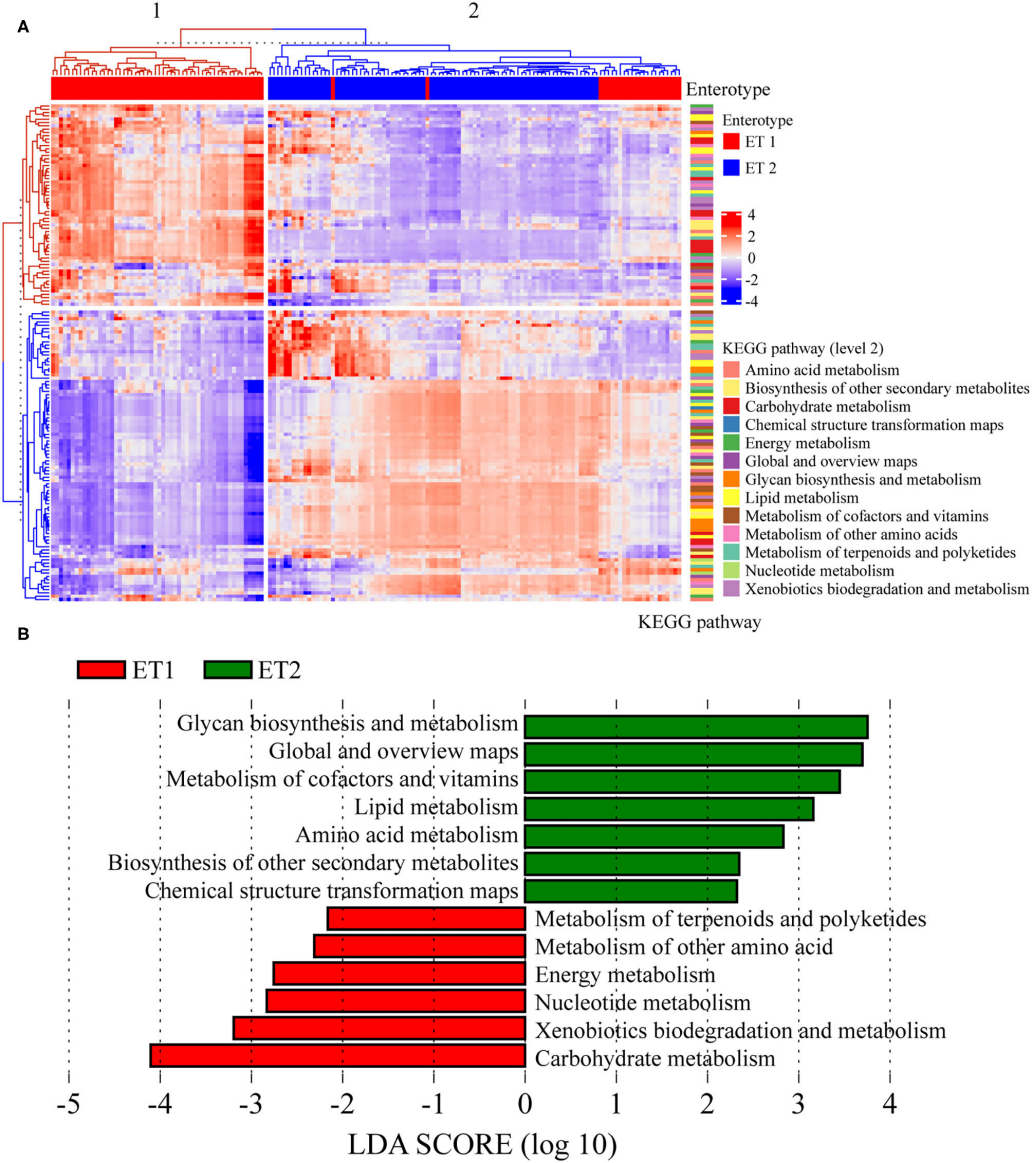
Prediction of the function of bacterial communities based on 16S rRNA sequencing. **(A)** The third level of the Kyoto Encyclopedia of Genes and Genomes (KEGG) pathway was shown in the heatmap. **(B)** Analysis for the KEGG pathway (level 2) using LEfSe.

Tax4Fun2 was used to infer functional biological pathways associated with the ETs in bacterial communities. We performed the KEGG analysis of the microbial contents of colon and fecal samples. Tax4Fun2 predicted a total of 7,201 KEGG Orthology (KO) genes from the microbial communities associated with the two ETs. In gut microbiota, the KEGG functions (pathway level 3) of rats in ET1 differed from those in ET2 (Figure 7A). Samples could be grouped into two clusters: cluster 1 and 2, which corresponded to ET1 and ET2, respectively. LEfSe analysis revealed significant differences in pathways in the two ETs. Glycan biosynthesis and metabolism, metabolism of cofactors and vitamins, lipid metabolism, amino acid metabolism, and biosynthesis of other secondary metabolites enriched in the ET2 group and metabolism of terpenoids and polyketides, metabolism of other amino acid, energy metabolism, nucleotide metabolism, xenobiotics biodegradation and metabolism, and carbohydrate metabolism enriched in the ET1 group (Figure 7B).

**Figure 8:**
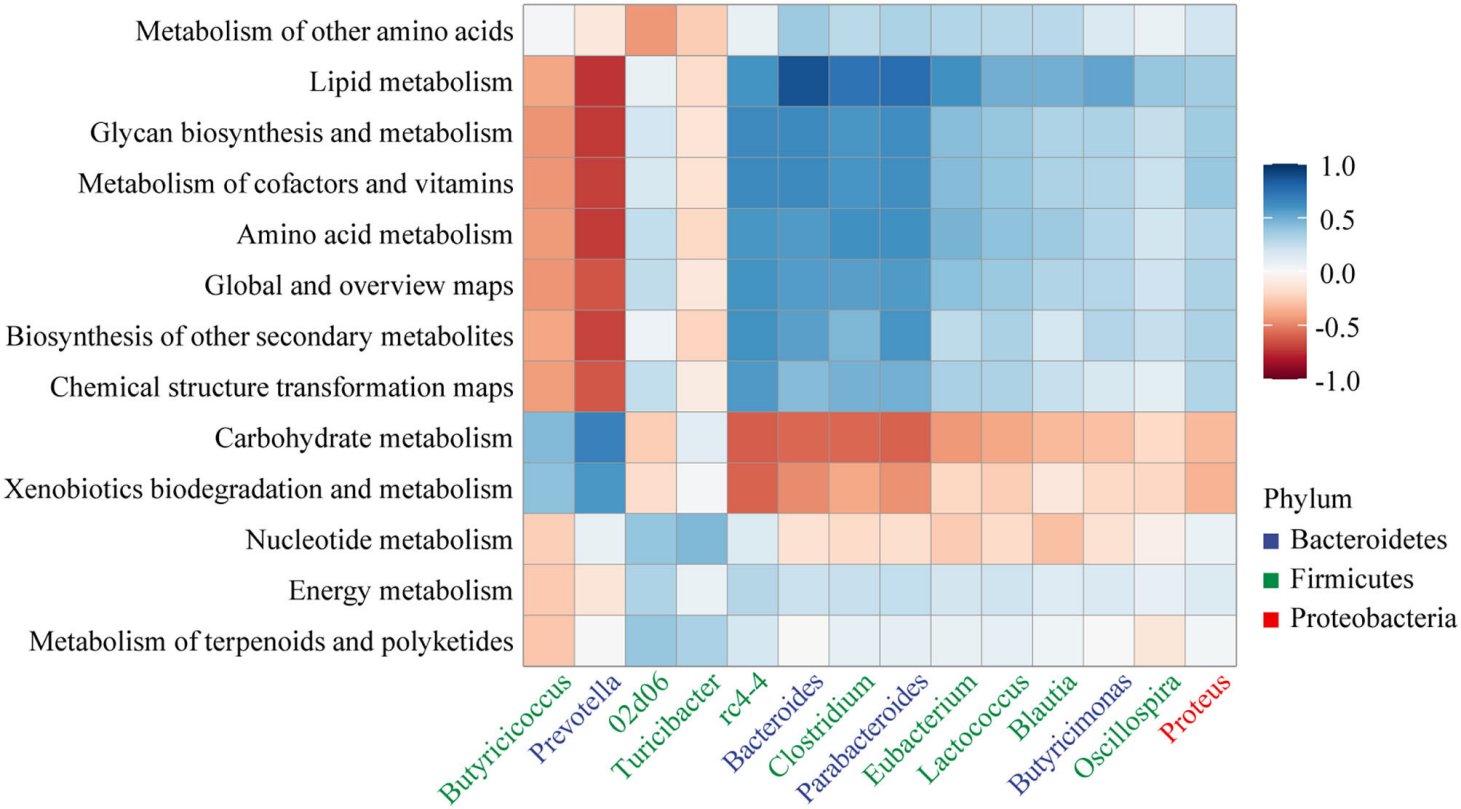
Heatmap of the Pearson's rank correlation coefficient between the KEGG pathway (level 2) and gut microbiota.

Correlation between genus (relative abundance >1%) and metabolic pathways was analyzed as shown in Figure 8. *Bacteroides, Clostridium*, and *Parabacteroides* showed a strong positive correlation with lipid metabolism (*r* > 0.7, *p* < 0.001). Amino acid metabolism, glycan biosynthesis and metabolism, lipid metabolism, and metabolism of cofactors and vitamins showed a strong negative correlation with *Prevotella* (*r* < −0.7, *p* < 0.001).

## Discussion

Rodent models of HFD-induced obesity are widely used in a plethora of studies, as they mimic human obesity and gut microbiota dysbiosis as a result of consuming fats (31). In this study, the composition of the initial gut microbiota was found to be similar to that of most mammalian gut microbiota with *Bacteroidetes* and *Firmicutes* as dominant bacterial groups (32). HFD perturbs the gut microbiota and develops a stable microbial overwhelmed by low levels of *Prevotella* and a high level of *Akkermansia, Bacteroides, Blautia*, and *Clostridium*, which has negative implications on host phenotype, as an example, this microbial consortia lead to an increase in body weight (33). As a major genus in the phylum *Bacteroidetes, Prevotella* has a direct correlation with die, as more consumption of carbohydrates and fiber leads to an increase in *Prevotella* content whereas fat and amino acids reduce it (34). In agreement with previous findings, we found a higher level of *Prevotella* in the NC group rats compared with the HFD group rats. Gut microbiota changes in rate as a result of BB-12 intervention that could be divided into two phases: At 0–3 weeks, structure of the gut microbiota was similar to the microbiota structure of the NC group rats whereas structure of the gut microbiota was similar to the microbiota structure of the HFD group rats from weeks 4 to 8. Some studies have shown that long-term HFD can alter the overall gut microbiota and shape the structure of the gut microbiota (35). So, it can be deduced the similar gut microbiota between the HFD group and the BB-12 group at weeks 4 to 8 may be due to the long-term HFD, which may be difficult for BB-12 to regulate. A higher abundance of *Prevotella* was observed in the BB-12-supplemented group compared to the HFD group from weeks 0 to 3. One important characteristic of *Prevotella* is to produce high levels of short-chain fatty acids (SCFAs), which against obesity by increasing energy expenditure in the liver (36, 37). So, increasing the abundance of *Prevotella* may be attributed to BB-12 supplementation as an effective strategy to improve obesity. In addition, some harmful bacterial contents were also decreased in BB-12-fed rats compared with HFD rats at 0–3 weeks. For instance, *Blautia*, which is associated with obesity as a result of its ability to produce acetate (38, 39), *Clostridium difficile*, which leads to healthcare-associated diarrhea by producing virulence determinants (40), and *Bacteroides*, which is associated with an increasing risk of obesity (41). At the end of the feeding period, the community structure of gut microbiota in the BB-12-fed rats group was similar with the HFD group rats and the similar community structure may be shaped by a long-term HFD induction (42). We also found some beneficial bacteria being enriched in rats of the BB-12-fed group. For example, *Eubacterium*, which is known for producing butyrate and can attenuate diet-induced obesity, insulin resistance, and hyperlipidemia (43, 44), *Parabacteroides* species, which have been reported as SCFAs-producing bacteria and exert anti-inflammatory effects (45), and *Bifidobacterium*, which is beneficial in general health and well-being (46). All the three genera enriched in the BB-12-fed group rats can help to improve obesity and its related disorders. Abundance of *Akkermansia* increases in the HFD group rats in agreement with previous studies (47, 48) and the increased *Akkermansia* abundance induced by HFD may help to improve inflammation (49). Most studies have shown a positive association of *Akkermansia* with the improvement of metabolic disorders (32), especially, *Akkermansia muciniphila*, which is known as one of the next generation probiotics (50). In fact, the relationship between *Akkermansia* and obesity is complicated and there are many other factors, such as aging and health status, which affect the abundance of this species in a gut (51). Thus, more mechanistic studies are needed to study the role of this species in obesity.

Enterotypes is one of the important microbial clustering techniques to investigate gut microbiota. ET1, ET2, and ET3 have been identified as the three dominant ETS in the human gut by metagenome sequencing with the dominance of *Bacteroides, Prevotella*, and *Ruminococcus*, respectively (28). Studies have shown that ETs are strongly related to long-term dietary habits (52). In this study, we found that the ETs are diet-dependent factors. Microbiota of rats belonging to the NC group with control diet was regarded as ET1 with *Prevotella* as the dominant genus and rats of the HFD groups had ET2 microbiota with *Akkermansia* as the dominant genus. BB-12 supplementation protected the *Prevotella*-dominant ET to against HFD induced. Recent studies have shown that *Prevotella*-dominant ET shows greater responses than other ETs for the administration of probiotics including the greater reduction of obesity-related markers (53). This suggested that ET was able to maintain a fine interaction with BB-12 supplementation and showed a correlation with HFD-induced obesity.

The F/B ratio proved to be another mean to instigate the gut microbiota of rats. Most studies have shown that the host adiposity is related to an increase in the ratio of F/B because *Firmicutes* and *Bacteroidetes* play an important role in metabolism of carbohydrates, lipids, and amino acids (10). In this study, a weak but significantly positive correlation was found between BW and F/B ratio. Besides, it was higher in the HFD group rats than the NC group rats due to the higher abundance of *Bacterioides* in the NC group. Differences in F/B ratio also existed in the two ETs; the ET2 group had higher F/B ratio than the ET1 group. Previous studies have shown that the F/B ratio is higher in obese individuals than in lean individuals (54). Thus, the changes in ETs corresponded well with changes in the F/B ratio.

Understanding of disruption of the gut microbiota functions is important in the pathogenesis of obesity. 16S rRNA functional annotation and correlation analysis have shown that bacteria from the phyla *Firmicutes* and *Bacteroidetes* are associated with carbohydrate metabolism, lipid metabolism, and amino acid metabolism. Studies have shown that gut microbiota can alter amino acids metabolism (aromatic and branched-chain amino acids metabolism), which is associated with insulin resistance and type 2 diabetes (55). Disturbance of lipid metabolism has also been associated with human obesity (56). In this study, we found a difference in the metabolic patterns of the two ETs. *Prevotella*-dominant ET has been proved to be associated with carbohydrate-rich diet (57). The same results have been achieved in this study, as carbohydrate metabolism was enriched in ET1 as well as it showed a strong positive correlation with *Prevotella* abundance. It is further illustrated that diet may be an influencing factor for the gut metabolism because of its influence on ETs and BB-12 supplementation provides protection of *Prevotella*-dominant ETs.

Overall, ET changed from ET1 to ET2 by HFD intervention and marked by a decrease in beneficial bacteria such as *Prevotella*. For antiobesity, it is necessary to suppress the transition from healthy to obesity as well as to improve from the obese state. BB-12 treatment ameliorated obesity in two different phases; in the first period (weeks 0–3), BB-12 protected the gut microbiota community structure to counteract obesity by promoting the growth of beneficial bacteria (such as *Prevotella*) and by decreasing the growth of harmful bacteria (such as *Clostridium, Blautia*, and *Bacteroides*), which clearly means a suppression of the transition from the healthy state to the obese state. An increase in alpha diversity and a decrease in BW were observed at week 3. In the second period (weeks 4–8), some beneficial bacteria were enriched (such as SCFAs-producing bacteria *Eubacterium* and *Parabacteroides* and probiotics *Bifidobacterium*) in the BB-12-fed rats group compared to the rats of the HFD-fed group, despite similar gut microbiota community structure. Our results provided further evidence of the role of BB-12 in maintaining gut microbiota and in ameliorating obesity through gut microbiota balance.

## Data Availability Statement

The datasets presented in this study can be found in NCBI-SRA under accession number PRJNA791415.

## Ethics Statement

The animal study was reviewed and approved by Center of Disease Control and Prevention, China.

## Author Contributions

KM contributes to the conceptualization, methodology, writing—original draft, and ad visualization. JG contributes to the conceptualization, resources, writing—review and editing, and funding acquisition. XW contributes to the resources, software, and data curation. XL contributes to the methodology and software. SG and TZ contributes to the writing—original draft. FS contributes to the writing—review and editing. YS contributes to the investigation, writing—review and editing, supervision, project administration, and funding acquisition. All authors contributed to the article and approved the submitted version.

## Funding

This study was funded by the National Key Research and Development Program of China (2019YFD0902003) and the National Natural Science Foundation of China (32101911).

## Conflict of Interest

The authors declare that the research was conducted in the absence of any commercial or financial relationships that could be construed as a potential conflict of interest.

## Publisher's Note

All claims expressed in this article are solely those of the authors and do not necessarily represent those of their affiliated organizations, or those of the publisher, the editors and the reviewers. Any product that may be evaluated in this article, or claim that may be made by its manufacturer, is not guaranteed or endorsed by the publisher.

## Supplementary Material

Supplementary Figure 1 Body weight and serum lipid levels. **(A)** The body weight changes during 8 weeks feeding period (mean ± SE), ^*^indicates a statistically significant difference (*p* < 0.05) between the HFD and NC groups; **(B)** Total cholesterol (TC), mmol/L; **(C)** Triglyceride (TG), mmol/L; **(D)** high-density lipoprotein cholesterol (HDL-C), mmol/L; **(E)** low-density lipoprotein cholesterol (LDL-C), mmol/L. ^*^*p* < 0.05, analysis in the *Wilcoxon* signed-rank test.

Supplementary Figure 2 Gut microbiota richness and diversity. 16S rRNA gene sequencing of 159 samples (143 fecal samples and 16 colonic contents samples) from 16 HMA rats were performed. Observed species and Chao1 indices reflect the richness of species, while he Shannon and Simpson indexes represent microbial alpha diversity.
