# Supplementary figures and images for: Bifidobacterium animalis subsp. lactis BB-12 Has Effect Against Obesity by Regulating Gut Microbiota in Two Phases in Human Microbiota-Associated Rats
Source: Front Nutr. 2022 Jan 10;8:811619. doi: 10.3389/fnut.2021.811619 (PMC8784422; doi:10.3389/fnut.2021.811619)

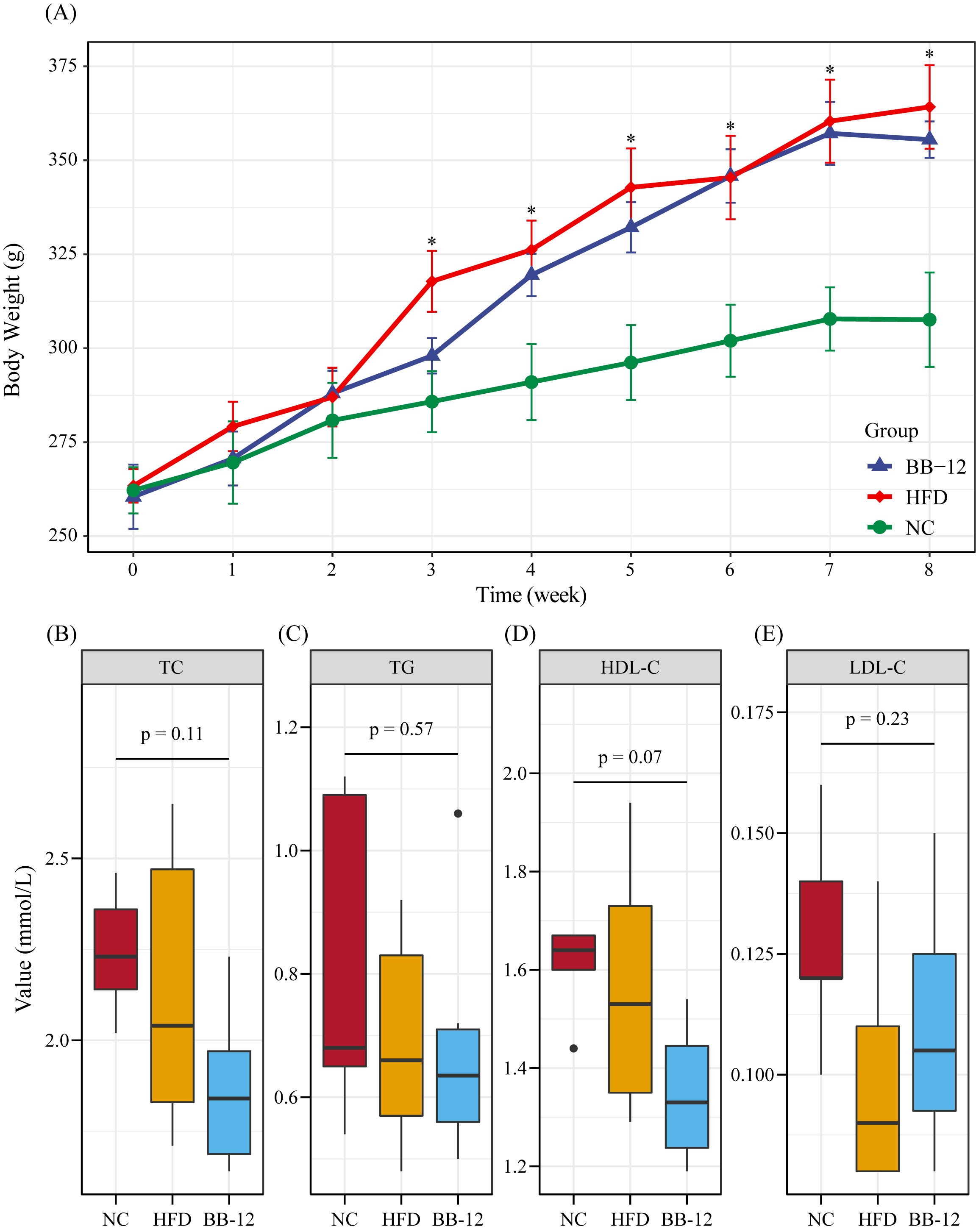

Supplement: Supplementary Figure 1 — Body weight and serum lipid levels. (A) The body weight changes during 8 weeks feeding period (mean ± SE), *indicates a statistically significant difference (p < 0.05) between the HFD and NC groups; (B) Total cholesterol (TC), mmol/L; (C) Triglyceride (TG), mmol/L; (D) high-density lipoprotein cholesterol (HDL-C), mmol/L; (E) low-density lipoprotein cholesterol (LDL-C), mmol/L. *p < 0.05, analysis in the Wilcoxon signed-rank test. [file Image_1.TIF]

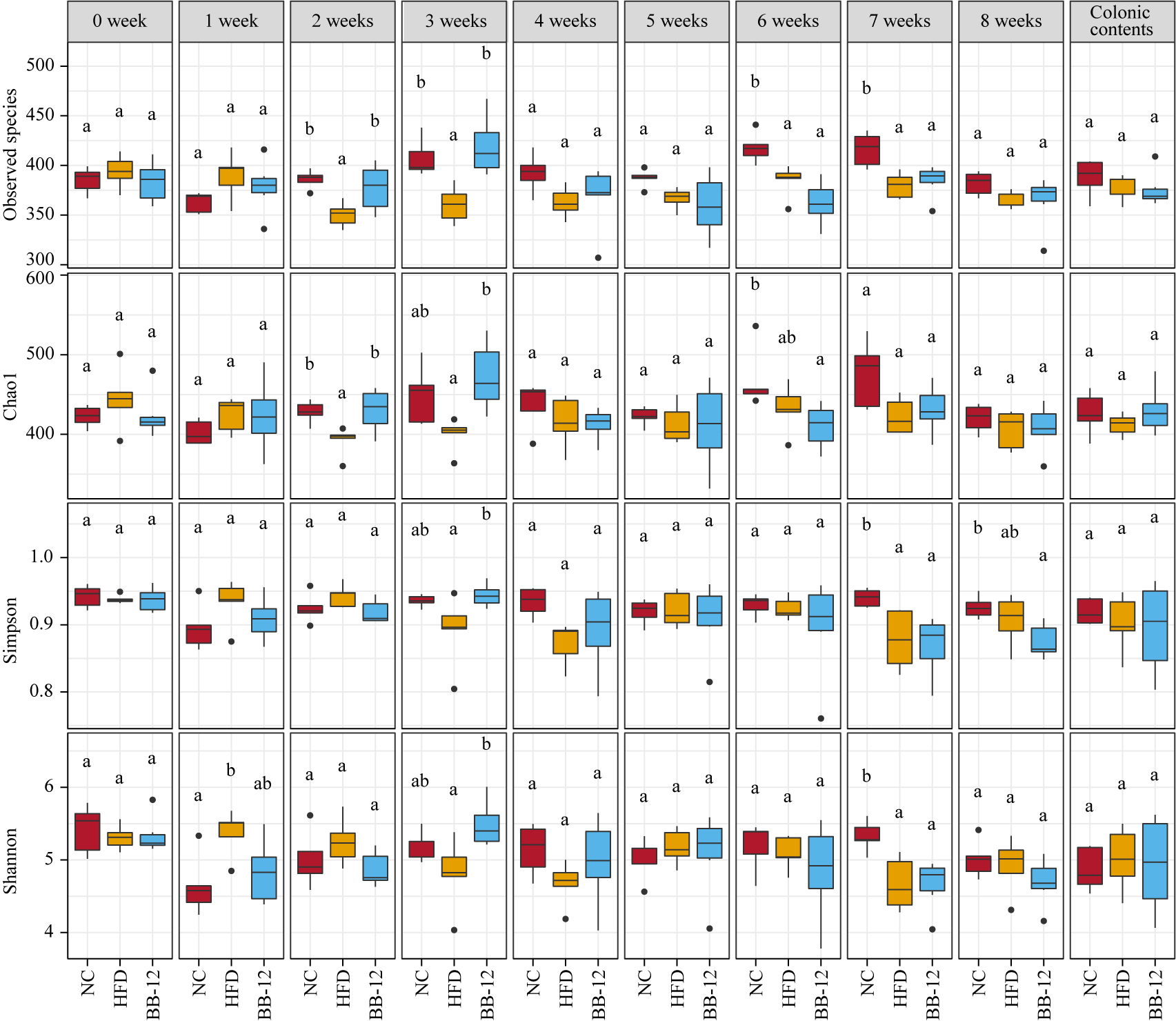

Supplement: Supplementary Figure 2 — Gut microbiota richness and diversity. 16S rRNA gene sequencing of 159 samples (143 fecal samples and 16 colonic contents samples) from 16 HMA rats were performed. Observed species and Chao1 indices reflect the richness of species, while he Shannon and Simpson indexes represent microbial alpha diversity. [file Image_2.TIF]
